# Sexual health counselling targeting girls and young women with female genital cutting in Sweden: mind–body dualism affecting social and health care professionals’ perspectives

**DOI:** 10.1080/26410397.2019.1615364

**Published:** 2019-06-11

**Authors:** Camilla Palm, Birgitta Essén, Sara Johnsdotter

**Affiliations:** a PhD Candidate, Faculty of Health and Society, Centre for Sexology and Sexuality Studies, Malmö University, Malmö, Sweden; Department of Women's and Children's Health (IMCH), Uppsala University, Uppsala, Sweden.; b Professor, Department of Women’s and Children’s Health (IMCH), Uppsala University, Uppsala, Sweden; c Professor, Faculty of Health and Society, Centre for Sexology and Sexuality Studies, Malmö University, Malmö, Sweden

**Keywords:** female genital cutting, female genital mutilation, sexual health counselling, discourses, migration, youth, Sweden, sexuality, body

## Abstract

Female genital mutilation (FGM), also referred to as female genital cutting (FGC), has become the subject of an intense debate exposing tensions between varying cultural values about bodies and sexuality. These issues are brought to the fore in settings where professionals provide sexual counselling to young circumcised women and girls in Western, multicultural societies. This article is based on interviews and focus group discussions with professionals in social and healthcare services. The aim of this study was to examine how professionals reflect upon and talk about sexuality and the promotion of sexual wellbeing in young circumcised women and girls. Policy documents guide their obligations, yet they are also influenced by culture-specific notions about bodies and sexuality and what can be called “the FGM standard tale”. The study found that professionals showed great commitment to helping the girls and young women in the best possible way. Their basic starting point, however, was characterised by a reductionist focus on the genitalia’s role in sexuality, thus neglecting other important dimensions in lived sexuality. In some cases, such an attitude may negatively affect an individual’s body image and sexual self-esteem. Future policy making in the field of sexual health among girls and young women with FGC would benefit from taking a broader holistic approach to sexuality. Professionals need to find ways of working that promote sexual wellbeing in girls, and must avoid messages that evoke body shame or feelings of loss of sexual capacity among those affected by FGC.

## Résumé

La mutilation sexuelle féminine (MSF), aussi appelée excision, est devenu un sujet de débat intense qui expose des tensions entre différentes valeurs culturelles sur les corps et la sexualité. Ces questions sont mises en avant dans des environnements où les professionnels donnent des conseils sexuels aux femmes et filles excisées qui vivent dans les sociétés multiculturelles occidentales. Cet article est fondé sur des entretiens et des discussions par groupe d’intérêt avec des agents des services sociaux et des soins de santé. Le but de cette étude était d’examiner comment ces professionnels réfléchissent à la sexualité et à la promotion du bien-être sexuel des jeunes femmes et filles excisées et comment ils en parlent. Des documents directeurs guident leurs obligations, pourtant, ils sont aussi influencés par des notions propres à leur culture sur les corps et la sexualité et ce que l’on peut appeler « l’histoire type de la MSF ». L’étude a révélé que les professionnels s’efforçaient d’aider les filles et les jeunes femmes de la meilleure façon possible. Leur point de départ de base était néanmoins caractérisé par une optique réductionniste sur le rôle des organes génitaux dans la sexualité, ce qui les amenait à négliger d’autres dimensions importantes dans la sexualité vécue. Dans certains cas, une telle attitude peut influer négativement sur l’image corporelle d’un individu et son estime de soi sexuelle. Il serait bon que les futures décisions politiques dans le domaine de la santé sexuelle des filles et des jeunes femmes excisées adoptent une approche holistique plus large de la sexualité. Les professionnels doivent trouver des méthodes de travail qui favorisent le bien-être sexuel des filles, et il leur faut éviter les messages qui évoquent la honte du corps ou des sentiments de perte de capacité sexuelle chez les excisées.

## Resumen

La mutilación genital femenina (MGF), también conocida como ablación genital, es tema de un intenso debate que expone las tensiones entre diversos valores culturales sobre el cuerpo y la sexualidad. Estos asuntos se ponen de relieve en contextos donde profesionales brindan consejería sexual a mujeres jóvenes y niñas circuncisas en sociedades occidentales multiculturales. Este artículo se basa en entrevistas y discusiones en grupos focales con profesionales en servicios sociales y sanitarios. El objetivo de este estudio era examinar cómo los profesionales reflexionan y hablan sobre sexualidad y la promoción del bienestar sexual en mujeres jóvenes y niñas circuncisas. Los documentos de políticas guían sus obligaciones; sin embargo, también influyen en ellos nociones culturales específicas sobre el cuerpo, la sexualidad y lo que se puede denominar “el cuento estándar sobre MGF”. El estudio encontró que los profesionales mostraron un gran compromiso para ayudar a las mujeres jóvenes y niñas de la mejor manera posible. Sin embargo, su punto de partida básico estaba caracterizado por un enfoque reduccionista en el rol de los genitales en la sexualidad; por lo tanto, hacía caso omiso a otras dimensiones importantes de la sexualidad vivida. En algunos casos, esa actitud podría afectar negativamente la imagen corporal y autoestima sexual de una persona. Para formular futuras políticas en el campo de salud sexual de mujeres jóvenes y niñas con MGF, sería útil aplicar una estrategia holística general con relación a la sexualidad. Los profesionales deben encontrar maneras de trabajar que promuevan el bienestar sexual de las niñas, y deben evitar transmitir mensajes que evoquen humillación corporal o sentimientos de pérdida de capacidad sexual entre las personas afectadas por MGF.

## Introduction

Female genital mutilation (FGM), also referred to as female genital cutting (FGC), has become the subject of intense debate, exposing tensions between varying cultural values about bodies and sexuality. On the one hand, FGC is cherished as a meaningful intervention among women in many groups upholding the practice,^1–3^ yet it is perceived as unacceptable and unlawful in Western countries receiving immigrants from regions where FGC is customary. This poses specific challenges for professionals in these countries providing care and counselling to girls with FGC. Studies have shown that being confronted with stigmatising attitudes towards one’s body may impair body image in women and girls with FGC.^1,4^ Increased attention is being placed on how professionals can provide empirically grounded, sensitive, and holistic care and support for girls and women with FGC without contributing to stigmatisation and impaired self-esteem.^5,6^

Sexual health counselling is an arena in which norms and values about sexuality, body, and health are communicated and negotiated, and where both the provider and the recipient enter the conversation with their own specific set of ideas, experiences, and assumptions.^7^ The purpose of this study was to examine how professionals in social and healthcare services reflect upon and talk about sexuality and the promotion of sexual wellbeing in young circumcised women and girls. Our key question was “How are dominant ideas about FGC and sexuality played out in practice, and how do professionals handle potential tensions from conflicting conceptualisations of bodies, sexuality, and FGC in encounters with girls and young women with FGC?”.

In this paper we will demonstrate that professionals we interviewed tend to employ a genitally focused approach in their efforts to promote sexual wellbeing in girls and young women with FGC, often leading them to conclude that FGC erases or at least reduces sexual and bodily functionality. This approach adheres to a particular, Western, image of what constitutes a functional body and sexuality. We argue that strategies for promoting sexual wellbeing within a Western cultural paradigm might be effective if a girl shares the same cultural understanding. In encounters with girls and young women that do not share these ideas, however, such an approach, if employed exclusively and not in a holistic manner, might work counterproductively and risk imposing feelings of incompleteness and dysfunction.

## The context

In investigations of beliefs, values, and motivations of people across the world by the World Values Survey (WVS), Sweden stands out as an extreme example of individualism, secularism, and gender equality, not least when it comes to issues related to sexuality.^8^ The WVS makes explicit that values are culture-specific: that is, people’s notions of what is “good” and “bad” regarding sexuality and bodies are affected by cultural norm systems situated in a certain time and place.^9^

Since the early 1990s, the Swedish government and official bodies have run programmes aimed at ending FGC, including adoption of guidelines and action plans, as well as media campaigns and awareness-raising efforts targeting immigrants and professionals in childcare, schools, healthcare, and social services.^10–12^ These reflect international political consensus that the traditional practices of FGC constitute a violation of human rights and imply serious physiological, psychological, and sexual health threats to girls and women.^13,14^ Currently, professionals are required to provide both prevention and support in relation to girls and women with FGC.^12^ Support may include counselling in matters related to health and sexuality, or identifying medical conditions and finding appropriate treatment, or referring the girl or woman to a specialist.^12,15^ Preventive measures, stressed in the action plan,^12^ focus on attitude change, information about the legal prohibition of FGC, and identifying girls potentially at risk and reporting suspected child abuse. Professionals need to navigate these manifold undertakings, finding a balance between working to promote wellbeing among those who have already gone through the procedure without condoning the practice.

In a wider context, campaigns, national policies, and public media embed culture-specific ideas about sexuality, body and health, including powerful messages labelling women with FGC as sexually and bodily imperfect.^12,13,15,16^ The notion of destroyed sexuality is common in depictions of FGC in newspapers, journals and best-selling novels. Such descriptions of FGC have variously been discussed as the “FGM fantasy”^17^ or “the standard tale” about FGC.^18,19^ A prime example is Dirie & Miller’s bestseller *Desert Flower*, which has been influential in shaping narratives about FGC and its possible impact on sexuality. In Dirie and Miller, it is asserted that “the most minimal damage is cutting away the hood of the clitoris, which will prohibit the girl from enjoying sex for the rest of her life” (p. 218).^20^

Statements about destroyed sexuality, as found in “the FGM standard tale”, are not supported in academic literature. Most research on sexuality after FGC shows that even though some women need care for negative consequences from FGC, women with FGC have sustained capacity to feel sexual pleasure and sexual wellbeing.^5,6,21–23^ Further, in the countries of origin, notions of FGC as disfiguring the body or damaging sexuality are not common. Instead, genital modifications are typically associated with values such as enhanced femininity, adherence to aesthetic ideals, and improved status as a woman.^1,3,18,23,24^ Research shows that psychological expectations play a determinant role for sexual wellbeing: anxiety over one’s body or the outcome of sexual activity are major influences on sexual inhibition.^25,26^ This has important implications for professionals’ encounters, especially with sexually inexperienced women, as they are in a state of developing their own sexual self-image; young women with FGC who are resident in Western countries live in a society that is openly opposed to the practice and tells them that they are “mutilated” and deprived of their ability to ever enjoy sex. Such messages may thus negatively affect body image and sexual self-esteem.

## Theoretical framework: a holistic approach to sexuality

This article adopts a social constructionist theoretical approach to sexuality,^27^ which emphasises that notions of sexuality are influenced by cultural norms, discourses, and social and political practices specific to their sociocultural setting.^9,27,28^ Accordingly, conceptualisations of sexuality cannot be separated from their sociocultural context. This approach allows us to recognise that both providers and recipients carry with them beliefs about what is “natural”, healthy, good or bad, acceptable or repugnant, and that these beliefs are shaped by the cultural repertoires available to them.

The dominant Western understanding of sexuality has been informed by the pioneering model of Masters and Johnson, *The Human Sexual Response Cycle*.^29^ This model identifies four critical events in human sexual response: desire, arousal, orgasm, and resolution, and it has been used as a framework for understanding and measuring sexual dysfunctions in men and women (e.g., in the fifth and current version of *Diagnostic and Statistical Manual of Mental Disorders* [DSM-5]).^30^ It describes several physiological responses occurring during sexual excitation such as swelling of genital tissue and changes in heart rate.^29^ This understanding of sexual sensations as a genital event relies on a biomedical understanding of sexuality, giving priority to physiological processes for experiences of desire and sexual pleasure.^28^ Accordingly, processes that interfere with one of the stages have been interpreted in terms of “dysfunction”. The model has been criticised for placing too much emphasis on physiological aspects, and for not taking into consideration the importance of psychological, sociocultural, or relational aspects of human sexuality,^31^ and for not acknowledging the several pathways to female orgasm.^32^ Despite these theoretical and empirical shortcomings, the biomedically oriented approach holds a prominent position when it comes to defining sexual problems in the West.^33,34^

In this article, our analysis is informed by a holistic approach to sexuality, one which does not ignore the biomedical perspective, while also considering sexual self-concept/self-esteem, body image, sexual self-schema, and sexual relations as important aspects at the core of lived sexuality.^35^

## Methods

The empirical data in this article are based on three focus group discussions (FGDs) and 12 individual semi-structured interviews with a total of 20 professionals. Included were school physicians, school nurses, and nurses in primary care and residential childcare, welfare staff in school and social services, as well as health educators. All had experience with providing sexual health counselling, including FGC information aimed at girls and young women.

Participants were approached either by email sent to professionals in institutions that worked with immigrants, such as schools with newly arrived young people, through personal encounters at FGC-related seminars or lectures, or through previous contacts. Among the participants, one nurse had taken a university course in sexual health care and one nurse held a master’s degree in public health; others had no special training in sexual health issues. All were women, aged 26–63 years; none had a background in a region where FGC is customary. Three participants described themselves as non-Western. Participants had been in their profession for 3–20 years, and some had limited experience with counselling in relation to FGC while others had more. Professionals worked with girls in secondary and upper secondary school, aged 13–21 years. A few worked only with minors whereas most worked with both minors and women over 18 years of age. The word “girls” will hereafter be used to refer to the young people the professionals worked with, even though this does not exclude girls over 18 and thus adults by definition.

Interviews were conducted by the first author from 2016 to 2018. FGDs comprised three individuals each and lasted 1.5–2 h. Individual interviews lasted one hour on average except one that was interrupted after 20 min due to unforeseen circumstances. Many participants were interviewed on several occasions, often both in person at the professionals’ workplace and by telephone, which allowed for further elaboration of themes from the previous interviews. One professional participated in both an FGD and an individual interview.

A basic interview guide was used and focused on strategies used in encounters in relation to FGC in general and in relation to sexual health matters. It also included questions about the participants’ views of situations they thought had worked well and situations they had found challenging in providing care or support to girls.

The interviews were digitally recorded and transcribed together with notes from interviews conducted in informal contexts. Interviews were conducted in Swedish and the quotes have been translated by the authors. Drawing on qualitative analysis techniques, we searched for sub-themes and overarching themes^36^ in relation to sexuality and strategies for promotion of sexual wellbeing. The empirical data were interpreted in light of questions that arose from the social constructionist framework emphasising how people talk about, negotiate, and construct meaning on the sexual body and its capacity.^27^ In the interpretative process, a particularly strong overarching principle was identified, around which all other categories relating to sexual health promoting strategies seemed to be organised, namely a body-oriented approach to sexuality. We considered this particular aspect would add important perspectives to existing research on sexual health counselling and FGC, and thus we paid extra attention to this theme in re-readings of the transcripts.

Informed consent was obtained from all participants. Interviewees are referred to by numbers and information about profession, which provides some context while safeguarding confidentiality. This study has no formal decision on ethics approval. According to the Swedish Ethical Review Act the study does not require ethical vetting from an ethics review board (personal communication with a member of the board, 9 Nov 2017).

## Findings

### The (cut) genitals as the site for sexual sensation, dysfunction, and intervention

From the interviews with professionals, it was evident that staff were committed to do their best in helping girls with FGC. Professionals were attentive to identifying FGC-related health problems, and provided information about possible complications they thought were of relevance in relation to the procedure and about available paths for help and support. Upon individuals’ requests, professionals helped arrange appointments for deinfibulation, and many also reported accompanying girls to the hospital. Some, cautious about linking health problems to FGC, tried to refer the girl to a doctor or gynaecologist to obtain a proper medical assessment for genital or related concerns. Professionals also showed devotion to promoting sexual wellbeing in girls with FGC.

A salient feature in the interviews was that strategies promoted and ways of understanding girls’ situations were anchored in a biomedical model of health and sexuality, where priority is given to bodily functions, more precisely the genitals. These tendencies are discussed below, in relation to how professionals talk about and reflect upon sexuality in girls with FGC. First, we relate the professionals’ narratives to the discussion about “the FGM standard tale” before focusing on how these conceptions are played out in practice in sexual healthcare counselling with girls with FGC.

### FGC as interfering with sexual pleasure?

The idea that women have impaired sexual function after all types of FGC, as purported in the “standard tale”, was salient in our study. Many professionals were convinced that girls had lost their ability to enjoy sex as a result of FGC. Some did not relate loss of sexual capacity to a particular type of cutting, as expressed by one school welfare-officer (1):

“[with cutting] you take away all sexual pleasure for the woman.”

Others attributed loss of sexual sensation to procedures involving the clitoris, as asserted by a health educator (2):

“[if] they have done pricking on the girl’s clitoris, then you still might have destroyed parts of the nerves, because that’s what can happen.”*

These accounts testify to a Western tendency to view sexual sensation in circumcised women as something that could either be damaged or removed solely through cutting of the genitals, hence situating sexual desire and pleasure, or loss thereof, in the genitals. Yet, several school nurses stressed that they place emphasis on the size and extent of the clitoris to encourage girls about their ability to feel sexual pleasure, emphasising the inner genital structures that have been left intact after FGC. Here a nurse (3) at a residential home for children in state custody, who reported that she had extensive discussions about sexuality with all the girls she met:

“I usually tell them: you know, this thing about cutting in women, those who made that up were stupid. They thought that we only have a little tiny thing, but look how big it really is [referring to a 3D-clitoris]! So, we [women] can feel desire, that’s just a matter of being inventive, to try out and explore for yourself”.

Professionals stressed the importance of the clitoris for sexual sensation in line with the dominant discourse discussed above. Some of them, however, accentuated possibilities for sexual pleasure by focusing on the clitoral tissue that remains inside the body after FGC rather than seeing sexuality as irrevocably impaired by FGC.

Some participants had concerns about how sexual activities could be carried out in a pleasurable way if a girl had had her genitals altered, especially in the case of suturing. Lack of available positive examples of sex and FGC seemed to create insecurity about how to address the topic of sex when providing information or counselling to young women with FGC. Here, the professional’s available cultural framework did not provide sufficient guidance, as illustrated by a school nurse (4):

“[I wonder] how they [girls with FGC] think sex will be carried out. I think that’s really difficult when I have sex ed class and talk about how we look [down there], and I hand out pictures [of the genitals], and then I think ‘well, this is not how [intercourse] will be for you, you poor thing’ [said with sorrow in her voice] … Sometimes I think to myself, that I don’t even know how they can have sex [so how would they know]. You know I don’t really get it sometimes. How do you get through what has been sewn closed?”

The limitations of the current discourse as a point of reference for the professionals was evident in the following situation, in which a school nurse was confronted with new ideas: she had held a sex education class in which girls with FGC expressed worries about their future sexual life. The female Somali-speaking interpreter who assisted in the class offered to comment upon their worries, and she assured the girls that she herself as married and circumcised had a satisfying sexual life, and that she felt sexual desire and pleasure. The school nurse (5) commented upon the situation:

“I must admit I was totally convinced that it was impossible to feel pleasure after female circumcision. But now with [the interpreter] saying that it’s possible … [pause] I guess I have to think differently about it”. *(From notes)*

When confronted with a narrative that challenged her previous understanding of sexuality and FGC, she described herself as being in a state of re-evaluating previous understandings of FGC and its impact on sexuality, not fully knowing “what to believe anymore”. Whether or not the professionals had positive or negative notions about the possibilities for women with FGC to enjoy sex, these notions were focused on bodily aspects. How these ideas were played out in practice will be discussed below.

### Problems and solutions in the body

While a biomedical approach often led professionals to conclude that FGC without exception negatively interferes with sexuality and health, it was also within a biomedical discourse that solutions and strategies to promote girls’ sexual wellbeing were sought. While professionals were sometimes hesitant to communicate about sexuality, for fear of dampening girls’ expectations regarding their future sexuality, many reported engaging in discussions about sexuality with girls they encountered. Professionals balanced the dual tasks of not condoning a practice they thought of as harmful, unnecessary, or otherwise wrong, while at the same time trying to be sensitive and careful not to stigmatise girls who have already undergone FGC. Much of the support and care described by the professionals seemed to work well, as in this example recounted by a school nurse (6):

***School nurse in FGD:*** I remember a girl … she was so relieved because she was, when we talked about what the body looks like, biologically, and the clitoris, how it is embedded inside, because she thought it was all over, the sexuality, that she would never be able [to feel pleasure] … so she was relieved when she got information about the body … and at the same time, this girl I met, she had probably never fully approached her body but kept a certain distance to her [circumcision], that it [the circumcision] was no good and that she distanced herself and had never really [shows with her body language]…

***Interviewer:*** Touched and looked?

***School nurse in FGD:*** Yes. And there I think it would have been good to know if there was anywhere she could get [information about sexuality], depending on what type [of circumcision] she had, because the outcome may vary [depending on type]. There I felt a bit insufficient.

And while much of the support and care described by the professionals seemed to work well, the strong body-focused approach sometimes stood out as problematic. On some occasions, professionals seemed to transfer their own ideas about body and sexuality in counselling, without exploring the girls’ own conceptualisations. This often was reported in relation to promotion of deinfibulation surgery or in providing sex education, as in this example from a school nurse (7) recalling individual encounters she had had with girls:

“I showed [the girls] what the female genitals look like in the normal female body, and then after different types of circumcision. Then I told them that they cut off a bit of the clitoris and what the risks of that are, with the sexual feeling and everything.”

In a similar vein, many professionals took on the task of educating girls about what they thought could be negative sexual and reproductive health consequences resulting from FGC. Information about supposed problems appeared to be given regardless of how girls had presented their situation, which form of FGC they had described, or whether they had expressed having troubles in relation to FGC. Scenarios were often described along this line, here with examples from two school nurses (8 and 4):

“[I have asked] if they have problems … somatic problems, and then they usually say yes or no. Then I generally go on telling [them] that, well, later you might want to have sexual relations … then it can be that it’s a bit of a problem with sensation and that it might be difficult feeling pleasure and all.”

“What I said [to her] was that I have thought a lot about your problems with constipation and I think that it could be a symptom … when in fact you are circumcised and sewn closed. … But then if she insists that she’s not [circumcised], then it falls flat, you see? Then I can’t follow through with that.”

In other instances, girls more evidently rejected the professionals’ proposed solutions to their situation, for example regarding deinfibulation. Many professionals had tried without succeeding to encourage girls to undergo premarital deinfibulation. Professionals reasoned from a medical perspective when they argued that deinfibulation could help girls escape sexual or reproductive health problems. They argued that such surgery would prevent painful vaginal intercourse, severe menstrual pain, stagnation of menstrual blood, or blood accumulating in the vaginal cavity. Even though some reported having met girls who expressed relief when they received information about deinfibulation, many shared the experience that girls often rejected this opportunity, did not turn up at a doctor’s appointment even though arranged in agreement with the girl, or that girls were reluctant to listen to the information given. One school nurse (7) explained:

“No one has wanted it [deinfibulation], actually. To operate it, remove it [the FGC]. And they have been comfortable with this [FGC] since it’s a traditional, cultural thing. So, they didn’t want to get rid of it, and when I simply explained the disadvantages of keeping it compared to having it removed, and the advantages of having [the FGC] removed, they didn’t want to listen, and they felt that they still wanted to keep it because otherwise no men would want them.”

Despite the professionals’ efforts to clarify the advantages of performing deinfibulation, it appears that the girls most often had their own motives for not wanting the operation. The interviews suggest that the girls’ own perspectives, expectations, and feelings regarding their genital physiology were seldom explored. These examples demonstrate a tendency among professionals to highlight possible negative outcomes of FGC as a means to help girls recognise potential problems of FGC, or in order to encourage girls to link health problems with FGC, sometimes with encouragement to go through genital surgery in the form of deinfibulation. For some, discussing possible drawbacks of FGC was also a way of trying to promote negative attitudes to FGC in order to protect future daughters.

Too strong a focus on biomedical aspects, in combination with neglect on the professionals’ part to explore the girls’ own perspectives, appeared as a possible obstacle to developing understanding of the girls’ situation. Despite good intentions, what is communicated to girls with such framing is the conceptualisation of their genital status as undesirable or problematic, and this from a purely biomedical view. Consequently, the obvious risk here is that girls are coached into interpreting their sexual experiences and bodily sensations in terms of problems and displeasure, even though current research does not support statements about lost ability to feel sexual pleasure or, for example, constipation, as results of FGC.^21,37^

## Discussion

### A mind–body dualism approach

Mind–body dualism is the tendency within the Western biomedical paradigm to make a strict division between what is physical and what is mental, a tendency evident also in Western study. Our analysis of interviews with professionals suggests a widespread tendency to situate sexual sensations, dysfunctions, and suggested interventions exclusively in the (cut) genitals. In this discourse, both cut girls’ sexual and reproductive health problems as well as suggested solutions were primarily formulated in terms of physiological concerns; consequently they often seemed to neglect the girls’ own values, assumptions, and expectations around sexuality, body, and FGC. While the biomedical discourse usually led professionals to conclude that FGC always interferes with sexuality and body in a negative way, some used the biomedical discourse in efforts to promote girls’ sexual wellbeing by offering help for deinfibulation or encouragement regarding the capacity to experience sexual pleasure through emphasising which genital structures had been left intact. As mentioned, and similar to what has been found in an interview study among Swedish midwives,^38^ some of the professionals we interviewed assumed completely impaired sexual capacity to be a result of FGC. Other interviewees were not convinced about this, but instead asserted that girls still can feel sexual pleasure with the help from internal clitoral structures. It seems a discursive change is going on in Sweden in which the structure of the clitoris and its importance for female sexual pleasure have been stressed. This message has been displayed in newspapers, sex education, and public campaigning during the last couple of years.^39^ While we contend that this information is important and useful, nevertheless it situates sexual experience and pleasure exclusively in the genital area and thus fails to challenge the dominant reductionist view of sexuality.

The notion of the genitals as the primary site for female sexual and reproductive wellbeing constitutes a culture-specific understanding and framing of sexuality. It builds on a “mind–body” dualism approach in which priority is given to biomedical processes and reflects the age-long questions of where to situate health in relation to the “mind” or the “body”.^7,40^ This biomedical understanding is mainly focused on medical information about anatomy, physiology, and dysfunctional organs, and less on hard-to-measure psychological and sociocultural issues, which are crucial for lived sexuality.^7^ Given the prominent position that the biomedical paradigm has in informing Western understandings of sexuality^28^ and the Western construal of FGC,^21,41^ it is not surprising to find that among professionals, priority is given to genital physiology. A similar tendency can be seen when it comes to health care for women with FGC, some of whom experience that healthcare staff put too strong a focus on their genitals even when they seek help for other problems.^42–44^ The view among the professionals that FGC is a key determinant for ill health and dysfunction is also in accordance with how the issue traditionally has been approached in the research field, as reviewed elsewhere.^45^

That the genitalia, and what has happened to them, are given priority in care by the professionals towards girls with FGC, is not necessarily undesirable. It is also not wrong to encourage girls to go through deinfibulation. However, for these strategies to be empowering, they must be acceptable to girls and make sense to them in terms of how they understand “good” and “bad” as regards body, sexuality, and health.^7^ FGC in the girls’ natal countries is traditionally seen as “good” and as a way of inscribing identity (e.g. gender, religious or ethnic identity) and “the very notion of health is wrapped up in having FGC” (p. 53).^45^ Such meanings are shaped in a local cultural context and by personal experiences that might, or might not, have new meanings in diaspora.^45^ For girls who have adopted or already are holding cultural beliefs about FGC as undesirable or harmful to health and sexuality, biomedical approaches that promote deinfibulation or stress the importance of the clitoris’ inner structures for sexual enjoyment, can be helpful.^46^ However, for girls who do not hold these views of their bodies, there might be drawbacks to an approach that relies too heavily on physiological aspects.

A paradigm that systematically emphasises the role of the genitals in sexual response and arousal, presumes that any type of FGC will interfere with optimal functionality, so-called genital determinism.^47^ In such a paradigm, there is little room for being both cut, or “mutilated”, *and* functioning.^48^ The only possible way to enhanced sexual health within such a discourse lies in medical interventions that somehow “undo” the cutting (e.g., deinfibulation or reconstructive surgery of the clitoris) or focusing solely on what has been left “intact”. Girls and women who do not share these conceptions of FGC and sexuality, or who do not wish to further modify their genitals, still have to carry the weight of being labelled as disfigured, or “mutilated”, and dysfunctional.

As acknowledged in systematic reviews,^21,37^ biological aspects of sexuality are inseparable from social aspects.^40,45^ This situation presents a challenge in establishing the scientific “facts” about FGC and its impact on sexuality and health. The current discrepancy between well-meaning information about possible negative outcomes and the real-life experiences of girls and women with FGC, may thus undermine the legitimacy of information efforts.^49^ Furthermore, there is a risk of suboptimal care if the caregiver offers care based on what is known about a group at a group level instead of information solicited in the personal encounter with an individual girl.

Although overwhelmingly compelling, the biomedical paradigm has proven insufficient for describing people’s experiences of sexuality.^28^ While genitalia are usually central to sexual activity, recent discussions on sexuality recognise sexuality as multifaceted, where the role of the brain, relational aspects, sociocultural dimensions, and psychological expectations for orgasm and sexual satisfaction must be given stronger weight.^26,31^ Here too a genitally focused approach runs the risk of neglecting other aspects of importance to sexual wellbeing.^35^ A growing body of literature on genital self-esteem and its relation to female sexual function shows that body shame, anxiety over body appearance and a negative attitude towards one’s genitals undermine sexual wellbeing and the ability to enjoy sex.^26,50–52^ This broader approach to sexuality and sexual pleasure, in which it is acknowledged that discourses have a bearing on a person’s lived sexuality, accentuates the importance of exploring what discourses, ideas, expectations, and experiences girls are carrying regarding sexuality and body in relation to FGC, especially in situations of sexual counselling.

### Implications for practice

The discussion in this article has implications for both health policies and supportive interventions. For decades, there has been a global public discourse condemning the practice of FGC, and campaigning efforts aimed at eliminating FGC often highlight a multitude of possible adverse consequences of FGC, including assertions about erased sexual function. Few representations exist that provide girls or professionals with positive examples of how sex can be pleasurable also after FGC, although we provide an example of an exception in Box 1. On an individual level, too strong an emphasis on genital physiology, including negative psychosexual messages to girls that their ability to enjoy sex might have been destroyed, runs the risk of leaving girls with FGC with feelings of hopelessness regarding their own prospects of a joyful sexual life. Further, in healthcare and social care encounters, adherence to the dominant Western discourse in which sexuality is primarily described in terms of physiological concerns is likely to leave professionals with an inadequate understanding of an individual girl’s sexual health. Such an approach risks neglecting other aspects that are vital for sexual pleasure, in line with a more holistic view of sex. Although it is critical to educate these girls and young women, like all girls and young women, about genital anatomy, physical sexual response, and biomedical signs of genital health and ill health, it appears to be equally important to raise discussions about broader issues such as self-confidence, wellbeing, and quality of life.^52^

Box 1. **Example of sex education for women with FGC**

The Swedish Association for Sex Education (RFSU), a civil society organisation with great political influence in Sweden, has recently changed their information about FGC on their website after years of only publishing condemning text about FGC. RFSU now provides a positive example of how sex can be pleasurable also after FGC. Under the heading “Can you have sex as genitally mutilated?”, the information now reads: “It is possible to both masturbate and have sex with others. It is not true that the genitals lose all sensation after a genital mutilation, or that the ability to feel desire and pleasure has disappeared. The whole vulva is full of nerve endings and it can be pleasurable to stimulate it in different ways. If the tip of the clitoris, that is, the visible part, has been cut off and there is only scar tissue there, you can try to stimulate the surrounding area with fingers or with a vibrator to be able to reach the parts of the clitoris that remain inside of the body. In order to reach the clitoris inner parts, it can be good to use a more powerful vibrator and a wider base on the vibrator. If the clitoral glans has been cut off, it may also be that other parts of the body become extra sensitive and feel especially nice to caress or kiss – such as lips, throat, nipples or thighs.” (The Swedish Association for Sex Education, 10 April 2019) [our translation from Swedish].

## Conclusion

In this article, we have shown that a biomedical, genitally focused approach to sexuality and sexual health is prioritised over other aspects of sexuality in sexual health counselling with girls who have undergone FGC. Professionals seemed to neglect exploring the girls’ own experiences and perceptions of the situation, which may open up room for professionals’ projecting their own perceptions, albeit unintentionally. They align to a framework based in biomedicine, characterised by genital reductionism. This narrow focus might not be problematic if the interpretation of the girl’s situation is shared between them and supportive measures are accepted by the girl. We suggest, however, that some of the interventions might generate unintended consequences, in that they may negatively affect the girls’ body image and sexual self-esteem. Future policy making in the field of sexual health among girls and young women with FGC would benefit from taking a broader holistic approach to sexuality. Professionals need to find ways of working that promote sexual wellbeing in girls, and must avoid messages that evoke body shame or feelings of loss of sexual capacity among the those affected by FGC.

## Footnotes

* Pricking refers to the practice where a sharp object is used on the clitoris to induce a drop of blood, sometimes also referred to as “symbolic circumcision”.
